# Trueness comparison of various intraoral scanners and hybrid workflow for ceramic restoration

**DOI:** 10.1590/1678-7757-2025-0065

**Published:** 2025-08-18

**Authors:** Taksid CHARASSEANGPAISARN, Kritsaya SRIMAKHAJON, Suchanan SASIWILASAKORN, Korravit HANVIVATTANAKUL, Pemika SANGVICHIT, Kornpavee SIMAPRONCHAI

**Affiliations:** 1 Rangsit University College of Dental Medicine Department of Prosthodontics Pathum Thani Thailand Rangsit University, College of Dental Medicine, Department of Prosthodontics, Pathum Thani, Thailand.; 2 Rangsit University College of Dental Medicine Pathum Thani Thailand Rangsit University, College of Dental Medicine, Pathum Thani, Thailand.; 3 Rangsit University College of Dental Medicine Department of Operative Dentistry Pathum Thani Thailand Rangsit University, College of Dental Medicine, Department of Operative Dentistry, Pathum Thani, Thailand.

**Keywords:** Dental impression technique, Dental model, Digital technology, Dimensional measurement accuracy, Three-dimensional imaging

## Abstract

The scanned abutment file for the digital design of restorations can be either obtained directly using the intraoral scanner (IOS) or scanning the impression or the working model with the extraoral scanner (EOS). The trueness of the scanned file pertains to its effect on the accuracy of the restoration.

**Objective:**

This study aimed to compare the trueness of scan files from different intraoral scanners (IOSs) and the hybrid workflow using the E3 extraoral scanner (EOS) for ceramic restoration.

**Methodology:**

The model of the mandibular right first molar was prepared for the ceramic crown, and it was scanned with the EOS in reference Standard Tessellation Language (STL) file format. The following seven experimental groups were investigated. The IOSs—iTero Element 5D (IT), Trios 4 (TF), Medit i700 (MI), Primescan (PM), Virtuo Vivo (VV)—were directly scanned on the prepared model. The silicone impression of the prepared model was scanned with EOS (IS). The working model poured from the impression was scanned with the EOS (WS). The test STL file was trimmed and superimposed on the reference STL file for the trueness assessment using Geomagic Control X. The point deviation at the surface and margin of each group were compared. The mean deviation was calculated and statistically analyzed with One-way ANOVA (α=0.05). The minimum and maximum deviation of each area were also recorded.

**Results:**

Compared with the other groups, the impression scan group had a significantly greatest deviation (p<0.05) in surface (37.65±1.14 µm), margin (63.57±5.85 µm) and overall (50.61±3.28 µm). The WS group showed significantly greater deviation (p<0.05) in surface (23.93±1.20 µm), margin (46.18±2.00 µm) and overall (35.05±1.16 µm) than the IOS groups. In some IOS groups, the deviation was also significantly different (p<0.05).

**Conclusion:**

The IOS is recommended for obtaining the scanned file due to its lesser deviation when compared to the hybrid workflow. While statistical differences exist among IOSs, the clinical relevance of these differences appears minimal. If the IOS does not exist, scanning the working model is preferred over scanning impressions directly. However, further clinical validation studies are necessary to confirm this finding.

## Graphical abstract

**Figure:**
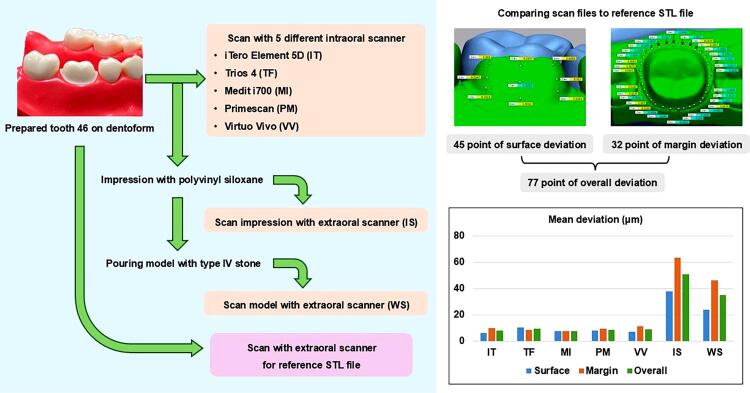

## Introduction

Intraoral scanners (IOSs) have been widely used in modern dentistry to facilitate digital workflows. A recent study revealed that about 78% of dentists across the world use IOSs in their daily practice.^1^ This might be due to the non-invasive, efficient, low-waste, and patient-friendly characteristics of IOSs compared to those of conventional impression techniques.^2^ IOSs offer numerous advantages including increased patient comfort, reduced chairside time, and improved clinician workflow efficiency when compared to conventional impression techniques, which often involve the use of large impression trays and impression materials.^3^ IOSs can capture precise digital impressions directly from the patient’s mouth, which not only has streamlined workflows, but also has enhanced the accuracy and quality of dental restorations.^4,5^

While conventional impression techniques have been used in daily dental practice for decades, they are naturally prone to errors, such as distortion, voids, and inaccuracies arising from material properties, technique variability, and patient factors,^6-12^ which could lead to fitting of the restoration. Even though the IOSs have many advantages over conventional impression, cost-effectiveness and effectiveness of treatment still need to be considered. Furthermore, the detection of deep margin of prepared teeth in gingival sulcus with IOSs is difficult, and scanning accuracy could be affected by moisture, i.e. saliva and bleeding.^13,14^ In addition to IOSs, digital impression files might also be obtained using extraoral scanners (EOS) scans on either the convention impressions or working models, which might be called “hybrid digital-analog workflow” or “hybrid workflow.”^15,16^

One of the relevant factors for choosing the IOS is the accuracy of the digital file. According to ISO 5725-1:2003, the accuracy consists of two terms: “trueness,” defined as the ability to accurately reproduce the geometry and surface details of the test object, and “precision,” defined as the ability to repeat the geometry and surface details of the test object under stipulated conditions.^17^

Numerous IOSs are currently available on the market. The cost of IOSs is also available in many ranges, each employing distinct technologies and methodologies for image acquisition,^1^ which include confocal microscopy, active wavefront sampling, structured light, and laser triangulation.^5^ Understanding the comparative trueness performance of these different technologies is crucial for clinicians to select the most appropriate intraoral scanner for their specific clinical applications. Previous studies have shown that different IOS technologies could obtain different digital impression accuracies with distance deviation.^18,19^ However, the study of point deviation at the surface and margin of the file has not been performed. This study aimed to compare the trueness of different intraoral scanners and the hybrid workflow for ceramic restoration. The null hypothesis of this study was that the trueness of STL files obtained using different IOSs and the hybrid workflow was not different.

The findings of this study can help clinicians optimize their digital workflow, ensuring the accuracy of ceramic restorations. By identifying the most reliable scanning technique, the study contributes to improved treatment outcomes, reduced number of procedures that need to be redone, and enhanced patient satisfaction in digital dentistry.

## Methodology

### Sample preparation

**Figure 1:**
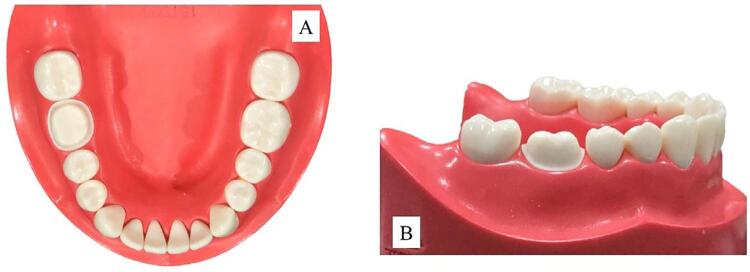
The prepared tooth 46 for ceramic restoration on a mandibular model, shown in an occlusal view (A) and a buccal view (B).

**Figure 2:**
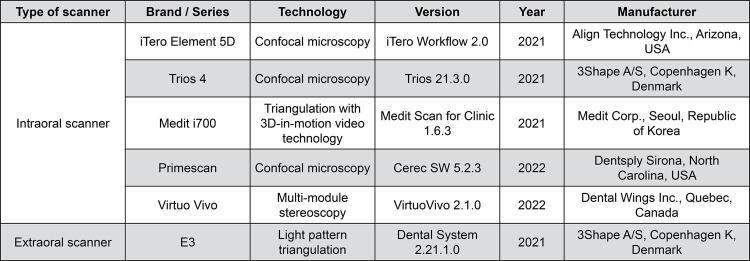
Details regarding the IOSs and EOS used in this study

The resin model of tooth 46, (Nissin Dental Product Inc., Kyoto, Japan) was prepared for the ceramic crown with a 1.2 mm heavy chamfer, a 0.5 mm supragingival finishing line, and a 1.5 mm occlusal reduction (Figure 1). Then the entire dental arch dentiform and prepared tooth were scanned using the extraoral scanner E3 for reference STL file (Figure 2).

**Figure 3:**
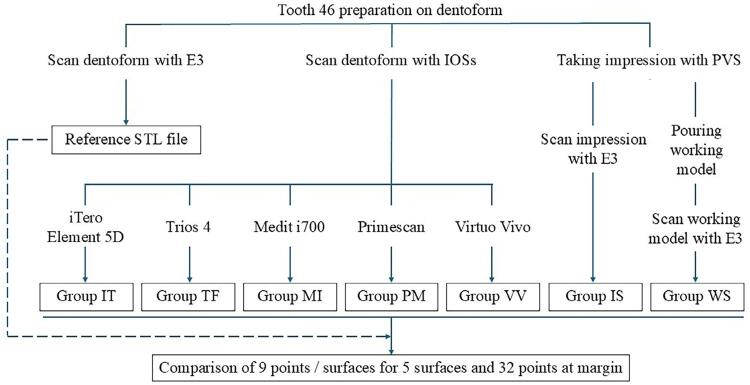
The experimental groups in this study.

**Figure 4:**
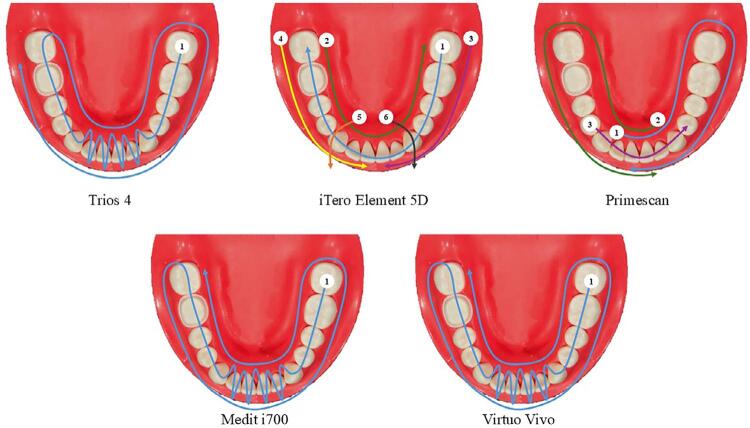
The scan strategies of each intraoral scanner.

The experiment was conducted in seven groups (n=10) as shown in Figure 3. Digital files pertaining to the iTero Element 5D (IT), Trios 4 (TF), Medit i700 (MI), Primescan (PM) and Virtuo Vivo (VV) groups were acquired using intraoral scanners (IOSs). The scan was performed by one experienced operator during the experiment. The operator was trained by the technical support team from each IOS distributor. The scanning strategies were performed following the manufacturer’s instructions (Figure 4).

After that, the full arch impression was taken using the double mix double impression technique with putty and light body polyvinyl siloxane (Silagum, DMG Chemisch-Pharmazeutische Fabrik GmbH, Hamburg, Germany). The putty silicone—prepared at a 1:1 ratio, measured with a two-point decimal digital scaler—was hand mixed. The light body silicone was mixed using a gun-type mixer at a 1:1 ratio. The working and setting times were in accordance with the manufacturer’s recommendations. The selected impression met the criteria of good margin detail, lack of excessive putty showing through, absence of voids, adequate extension, freedom from debris and extraneous material, adequate detail and proper recording of all structures. The impression was left for 24 hours prior to scanning with the EOS. The scan data were converted from negative surface to positive surface using the scanner software for the digital model (Group IS). After that, the impression was poured with type IV stone (Rocka, Noritake SCG Plaster Co., Ltd., Saraburi, Thailand), mixed at a ratio of 23 mL of water to 100 g of dental stone powder using a vacuum mixer to fabricate the working model. The working model was scanned with EOS to create a digital model (Group WS). All the acquired data were used for the trueness comparison with the reference STL file. The details of the IOSs and EOS were shown in Figure 1. All scanners used in this study were set up and calibrated according to the manufacturer’s recommendations before they were used for standardization.

### Trueness evaluation

The scanned STL files were exported and trimmed to specific areas, covering approximately the distal half of tooth 45 to the mesial half of tooth 47 using Autodesk Meshmixer (California, USA). All scanned data were imported into Geomagic Control X (North Carolina, USA) for the trueness assessment. The test STL files were superimposed on the reference STL file by initial alignment, after which the best-fit alignment algorithm was used for optimizing the fit across the entire surface.

**Figure 5:**
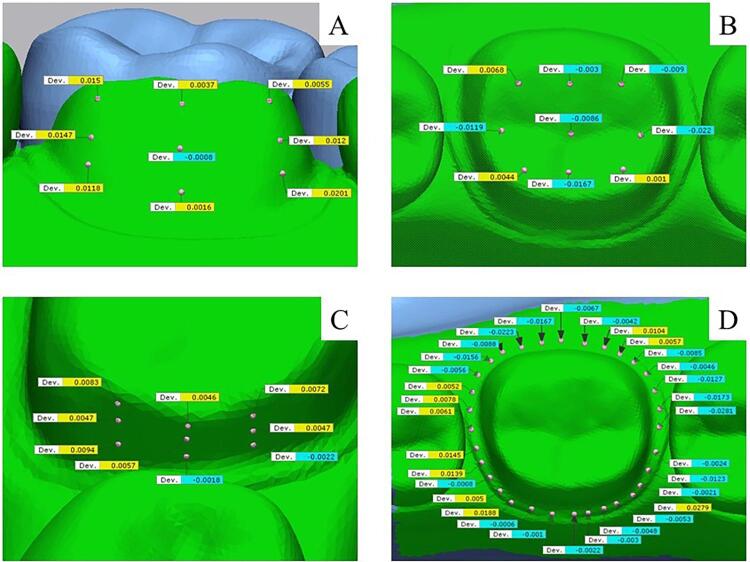
Representative areas of observation: buccal (A), occlusal (B), mesial (C) and margin (D) from group PM.

Measurements were taken for point deviation evaluation on both the “surface” and “margin” of the prepared tooth. For the surface deviation analysis, nine observation points were selected on five surfaces (occlusal, buccal, lingual, mesial, and distal), totaling 45 points. Following the division into thirds, the nine observation points were distributed over the tooth in nine sections. On each surface, the crown is divided into three parts and three directions: occluso-cervically, mesio-distally, or bucco-lingually.^20^ Then the points were located at the center of each section as the representatives of each section. For margin deviation analysis, the 32 observation points were also distributed circumferentially around the margin of prepared tooth (Figure 5). Due to the observed data including both positive and negative deviations, the absolute value of each deviation was used for analysis. The overall deviation was determined by calculating the mean absolute deviation across all 77 points, including both surface and margin.

### Statistical analysis

The single experienced operator was calibrated using intra-correlation coefficients (ICC) analysis. The ICC estimates and their 95% confident intervals were calculated using IBM SPSS version 26 (SPSS Inc, Chicago, IL), based on single rater, absolute-agreement, two-way mixed-effects model. The sample size per group was calculated using a G-Power analysis from the pilot study. The parameters for the G-Power analysis were: alpha error = 0.05, power = 0.95, effect size = 2.87. The G-Power analysis confirmed that the sample size in this study (n=10) was adequate.

The absolute deviation was calculated for mean deviation in terms of “surface,” “margin,” and “overall.” The deviation of the scanned files in each group (n=10) was assessed using the Kolmogorov-Smirnov (1-KS) test for normality. Subsequently, the data were analyzed using the one-way ANOVA with the Tamhane’s T2 test. The statistical analysis was performed using IBM SPSS version 26 with a 95% confidence level.

## Results

The ICC value was 0.881 (indicating good reliability), with the 95% confidence interval ranging from 0.818 to 0.922. This means that, based on statistical inference, it would be more appropriate to conclude that the level of reliability is on scale of “good” to “excellent”.^21^

The data of each group were analyzed, and the results showed a normal distribution in every group. In all test areas, the results indicated that the IS group had a significantly greater deviation (µm) than the other groups in all aspects (p<0.05), including surface (37.65±1.14), margin (63.57±5.85) and overall (50.61±3.28). The WS group also showed significantly greater deviation than the IOS groups (p<0.05) in surface (23.93±1.20), margin (46.18±2.00), and overall (35.05±1.16).

The deviation among the IOSs was also significantly different (p<0.05). Regarding surface deviation, IT (6.02±0.36) had the lowest deviation, and TF had the highest deviation (10.32±0.42), while MI (7.54±0.69), PM (7.95±0.71) and VV (7.14±0.76) were not significantly different (p>0.05). Regarding margin deviation, VV (11.23±1.25) showed the highest deviation but was not significantly different from IT (9.94±1.26) (p>0.05), while MI showed the lowest deviation (7.63±0.37), which was not significantly different from PM (9.22±1.23) and TF (8.61±0.82) (p>0.05). The overall deviation showed that MI (7.58±0.30) had the lowest deviation, which was not significantly different from IT (7.98±0.71) and PM (8.58±0.78) (p>0.05). In contrast to TF (9.47±0.54) which showed the highest deviation but not different from the VV (9.19±0.45) and PM (p>0.05). The results of all groups were shown in Table 1.

**Table 1:** Nomenclature, mean±SD, minimum and maximum deviation of each group in µm, including comparisons between groups.

Group	Surface		Margin		Overall
	Mean±SD	Min–Max	Mean±SD	Min–Max	Mean±SD
IT	6.02±0.36^A^	5.62–6.52	9.94±1.26^B,C^	8.44–12.62	7.98±0.71^A^
TF	10.32±0.42^C^	9.32–10.78	8.61±0.82^A,B^	7.35–9.40	9.47±0.54^B^
MI	7.54±0.69^B^	6.40–8.55	7.63±0.37^A^	6.97–8.11	7.58±0.30^A^
PM	7.95±0.71^B^	7.08–9.08	9.22±1.23^A,B^	7.42–11.91	8.58±0.78^A,B^
VV	7.14±0.76^B^	6.17–8.18	11.23±1.25^C^	8.7 –13.04	9.19±0.45^B^
IS	37.65±1.14^E^	36.13–40.13	63.57±5.85^E^	57.15–74.33	50.61±3.28^D^
WS	23.93±1.20^D^	22.37–26.23	46.18±2.00^D^	42.08–49.20	35.05±1.16^C^

* Group abbreviations: IT = iTero Element 5D, TF = Trios 4, MI = Medit i700, PM = PrimeScan, VV = Virtuo Vivo, IS = Impression scan using E3 extraoral scanner, and WS = Working model scan using E3 extraoral scanner.

** Different uppercase superscript letters within the same column indicate statistically significant differences (p < 0.05).

*** The statistical analysis of mean deviation was performed for each column individually^.^

## Discussion

From the results of this study, we can conclude that the null hypothesis is rejected due to the STL file obtained from the impression scan (IS) group having the lowest trueness, followed by the working model scan (WS). All the IOS groups demonstrated higher trueness than scanning from impression or working model, which will be discussed later.

The aim of this study was to compare the trueness of different IOSs and a hybrid workflow. The resin model was selected to minimize errors from clinical situations, such as movable soft tissue and saliva, which could create errors in the file scan.^14^ The margin of abutment preparation was placed 0.5 mm supra-gingivally to reduce the effect of light projection into the blind spot at the sulcus area. The double-mixed, double-impression technique was used to minimize the distortion of the impression.^6,10^ Trueness is defined as the closeness of agreement between the measured data and the reference standard. Therefore, a lower deviation indicates higher trueness, while a greater deviation reflects poorer trueness, meaning the scanned model is less accurate in reproducing the actual geometry of the prepared tooth.

The deviation of the scanned file can be evaluated as either “point deviation” or “distant deviation”. Many previous studies have observed distant deviation, which is calculated from the distance between two reference points on the X-Y-Z axis.^18,19^ However, studies on point deviation, which is observed at specific points in the scanned files, are still limited. Point deviation is appropriate for investigating specific areas of a file scan, such as surface deviation. When investigating abutment preparation for restorations, point deviation might be more appropriate than distant deviation. The surface deviation of the scanned file could affect the fit of the restoration on the abutment, leading to changes in cement thickness and ultimately affecting the longevity of the restoration.^22,23^ However, root mean square (RMS) was not used for deviation calculation in this study. RMS is calculated as the average deviation of all surfaces on scanned file, including the gingival and adjacent teeth, which were not within the scope of this study. Thus, point deviation was considered more appropriate for deviation analysis in this study.

The results of this study showed that IOSs achieved better trueness of the digital file compared to the IS and WS groups. The IS group had the greatest deviation and was significantly different from the other groups in all aspects. This is because the light from the scanner is projected onto the impression during the generation of the digital file and then reflected back to the photoreceptor device of the scanner.^5^ The impression surface was too challenging for the light to be projected onto and for data collection, especially in undercut areas, and areas where the light path was blocked by either the tray or the impression itself. This caused the scanner to scan several times to obtain the data in those areas. Even repeated scanning in the same position could help obtain missing details, but this also increases the error from each scan into the scanned file.

Scanning working models with lab scanner (WS group) also exhibited greater deviation than the IOS groups in all aspects. This might be caused by the procedures before scanning. The deviation could occur during many steps, such as the pouring method, the degree of stone expansion, and the quality of the impression material.^11,24^ In a previous study, the rate of type IV stone expansion was reported at approximately 0.11%.^12^ The deviation of the stone die was reported at approximately 79.1–96.8 µm.^25^ However, the deviation of the WS group was less than that of the IS group because the light could project directly onto the model surface with less scanning time. Thus, intraoral scanners enable direct digital capture of the intraoral environment, minimizing the potential for error and enhancing the reliability of impressions.

Recent review studies have shown that many factors can affect the accuracy of digital scanned files.^26,27^ Even though the IOSs used in this study have different image capturing technologies, tip sizes, and scanning strategies, these might not be factors that affect the trueness of the scanned files in this study. Based on overall deviation, the two IOSs with the highest trueness (MD and IT) were used with the high-definition mode for abutment scanning, which was supposed to be the factor that achieved the highest trueness among the IOS groups. The TF group exhibited the lowest trueness, but its deviation did not differ from the VV and PM groups. Although the deviation between the IOS groups was significantly different, this might not affect the clinical situation. The lowest and greatest mean deviations were reported at 7.58 and 9.47 µm for MD and TF groups, respectively. Thus, the difference was approximately only 2 µm, which might not be clinically significant. Considering the clinically acceptable marginal gap and cement space settings in clinical workflows, this difference is unlikely to affect clinical outcomes.^28,29^ Therefore, even though statistical variation exists, these deviations may not be clinically significant.

**Figure 6:**
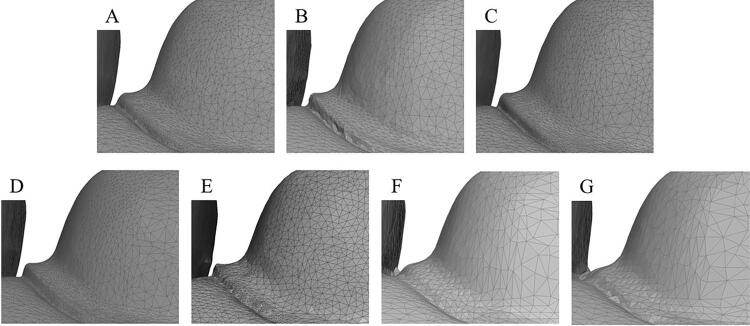
Mesh density of each group: iTero 5D Element (A), Trios 4 (B), Medit i700 (C), PrimeScan (D), Virtuo Vivo (E), impression scan (F) and working model scan (G).

In this study, the observation area was also divided into surface and margin. The deviation of the margin directly affects the marginal gap of the restoration. The deviation of the surface also affects marginal gap because the inner surface of the restoration could interfere the with restoration seating properly on the abutment.^30^ Based on a recent study, the clinical marginal gap of ceramic inlays obtained from digital impressions showed lower values than those from conventional impressions, at about 40 µm, which is similar to findings in this study.^31^ When comparing the deviation between the surface and margin, we found that the digital file deviated more at the margin than at the surface, except in the TF group. This might be because the margin has more curvature in the limited area than the surface. The structure of the digital file consists of polygons that form a mesh to create the surface. Mesh in curved area has a higher density than in smooth surfaces. Furthermore, the file scan from IOS groups showed higher density than those from WS and IS groups in both smooth surfaces and margin areas (Figure 6). Thus, the deviation of the margin could be observed more clearly than on the smooth surface. This result is in agreement with that of Park, et al.^32^ (2023), who reported that the deviation increased in the angle and curved area of the cavity. However, the surface deviation might be compensated by setting the cement gap in the range of 40–50 µm during the restorative design of the computer-aided design (CAD) program.^33^ A study by May, et al. ^28^ (1998) suggested that the clinically acceptable marginal gap for ceramic restorations should be less than 100 µm, reflecting advancements in materials and fabrication techniques. While the traditional threshold of 120 µm has been widely referenced, emerging evidence indicates that smaller gaps may be preferable for optimal clinical outcomes.^29^ Even though the impression is one part of restoration fabrication process, lower deviation in the scanned file could decrease the deviation of the final restoration.

The limitation of this study is that the observation was conducted *in vitro*, which might differ from clinical conditions. However, the benefit of *in vitro* research is that it limits the factors that might influence the results of the study, such as moisture, soft tissue displacement, and access to the working field. Future studies should imitate clinical conditions more closely, including moisture and accessibility of posterior teeth. The accuracy of fabricated restoration, occlusion involving the opposing teeth, and comparisons between different assessment methods, such as the closet distance, should also be further evaluated. Another limitation of this study is the absence of deviation maps, which could provide a more intuitive visualization of the discrepancies between the scanned and reference models, enabling better spatial understanding of the distribution. Future *in vitro* studies are encouraged to include such visual tools to enhance interpretation and clinical relevance.

## Conclusion

With the limitation of this study, the following statements are recommended for obtaining the digital scanned files for ceramic restorations:

*In vitro* study, scanning directly from the prepared model with IOSs demonstrated the least deviation in scan data. However, in clinical settings, factors such as saliva, soft tissue movement, and patient-related variables may affect accuracy.

Although statistically significant differences were observed among IOSs, the magnitude of these differences (e.g., within a few micrometers) is likely not clinically relevant and should be interpreted with caution.

If an IOS could not be obtained, scanning of the working model is recommended rather than impression scanning.

Due to this study conducted under controlled laboratory conditions, it is essential that further clinical validation studies be carried out to assess the reliability of these results in clinical situations.
